# Heart regeneration and the cardiomyocyte cell cycle

**DOI:** 10.1007/s00424-017-2061-4

**Published:** 2017-08-28

**Authors:** Michael Hesse, Armin Welz, Bernd K. Fleischmann

**Affiliations:** 1 Institute of Physiology I, Life & Brain Center, University of Bonn, Sigmund-Freud-Strasse 25, 53105 Bonn, Germany; 2 Department of Cardiac Surgery, Medical Faculty, University of Bonn, Sigmund-Freud-Strasse 25, 53105 Bonn, Germany; 3 Pharma Center Bonn, University of Bonn, Sigmund-Freud-Strasse 25, 53105 Bonn, Germany

**Keywords:** Regeneration, Cardiomyocyte, Heart, Transgenic animal models, Cell growth

## Abstract

Cardiovascular disease and in particular, heart failure are still main causes of death; therefore, novel therapeutic approaches are urgently needed. Loss of contractile substrate in the heart and limited regenerative capacity of cardiomyocytes are mainly responsible for the poor cardiovascular outcome. This is related to the postmitotic state of differentiated cardiomyocytes, which is partly due to their polyploid nature caused by cell cycle variants. As such, the cardiomyocyte cell cycle is a key player, and its manipulation could be a promising strategy for enhancing the plasticity of the heart by inducing cardiomyocyte proliferation. This review focuses on the cardiac cell cycle and its variants during postnatal growth, the different regenerative responses of the heart in dependance of the developmental stage and on manipulations of the cell cycle. Because a therapeutic goal is to induce authentic cell division in cardiomyocytes, recent experimental approaches following this strategy are also discussed.

## Introduction

At the beginning of the twenty-first century, cardiovascular diseases are still the number one cause of death in the industrial world. With respect to the heart, most of these diseases end up in congestive heart failure, caused by substantial loss of contractile tissue, volume, and/or pressure overload. The prevalence of heart failure in the adult population is estimated to be 1–2% in developed countries [51]. The most common aetiologies are ischemic coronary artery disease, systemic hypertension, structural valvular heart disease, and dilative cardiomyopathy. Despite improvements in treatment strategies, both hospitalization rates and mortality have remained high, especially in heart failure patients with reduced ejection fraction. For instance, the recent ESC-HF pilot study revealed a 12-month all-cause mortality of 17% for hospitalized and 7% for ambulatory symptomatic patients [46]. Death is due to worsening of congestive heart failure or to sudden death by ventricular arrhythmias. The only curative treatment strategy in end stage heart failure with reduced ejection fraction is orthotopic heart transplantation. However, this treatment is hampered by a large mismatch in recipients on the waiting list and available donors. In the meanwhile, implantation of left ventricular assist devices (LVADs), originally developed as bridge to transplantation, has become destination therapy for most patients. In Germany, LVAD implantation numbers per year increased to 915, whilst heart transplantation was below 300 in 2015 [7]. Results of LVAD therapy have improved significantly with regard to quality of life and survival, as 1- and 2-year survival rates have been reported to be 80 and 70%, respectively [38]. However, bleeding, thromboembolism and its complications (stroke and neurological deficits), pump failure, and drive line infections remain significant problems strongly affecting long-term survival rates [39, 72]. Thus, new regenerative approaches are clearly needed to restore loss of cardiomyocytes (CMS) and left ventricular function.

An approach that recently came into focus of several groups in the cardiac field is the induction of proliferation in resident, fully differentiated CMs. This is also motivated by the fact that cell replacement approaches appear difficult to translate into the clinics, because the ideal cell type has not been identified, stable long-term engraftment is difficult to achieve, and there appears to be a potential increase of ventricular arrhythmias after using pluripotent cell-derived CMs [13, 66]. Thus, the alternative idea is to stimulate surviving cardiomyocytes after a heart attack preferentially in the border zone to divide and to replace the lost myocardial mass. However, there are still conceptual questions, which need to be addressed until this strategy can be successfully applied: (i) Are there examples of cardiac regeneration by cardiomyocyte (CM) proliferation in the animal kingdom? (ii) How can a terminally differentiated CM be brought back into a proliferative state? (iii) Could massive proliferation of CMs be obtained without adverse effects onto pump function and electrical stability (arrhythmias). In this review, we will discuss these questions and try to shed new light on the manipulation of the CM cell cycle as a novel therapeutical approach.

## Regeneration, compensatory growth, and repair

First, it is important to define the term regeneration (from Latin regenerare “generate again”), which is used for all possible processes of tissue repair and set it apart from compensatory growth. These phenomena are based on completely different mechanisms of cellular response, which is especially evident when regeneration in amphibia and fish is compared to repair processes in mammals.

Salamander, newt, and zebrafish can regenerate complex structures such as limbs or fins by formation of a blastema either by activating stem cells or by dedifferentiation of somatic cells into stem/progenitor like cells (epimorphosis). In these cases, the original structure is reformed and thereby preserved, as has been described in complete regeneration of newt limbs [67], zebrafish fins [64], and axolotl brain [2]. On the other hand, liver regeneration in mammals after partial hepatectomy takes place by an increase of the mass of the remaining liver lobes. However, in this case, the original structure of the liver is not preserved, and the cellular composition was found to differ significantly from the unharmed organ. It has been reported that part of the liver grows back by hyperplasia, while there is also a hypertrophic component, which depends on the size of the removed liver mass (Miyaoka et al. 2012). During this phenomenon, hepatocytes increase their ploidy by endoreduplication. The role of this cell cycle variant was demonstrated by specific deletion of TRF2 in hepatocytes in mice, which blocks the ability of the cells to divide [41]. Even after the loss of this telomeric protein, the liver is able to regenerate solely by endoreduplication and hypertrophic cell growth, thereby restoring liver mass and function.

This form of repair is not regeneration per se, but compensatory growth as the original structure and cellular composition of the liver have been dramatically altered [41]. Thus, compensatory growth mechanisms are hypertrophy and/or hyperplasia. A second well-known example for compensatory growth is the repair process taking place after removal of one kidney. The remaining kidney increases in size by both hyperplasia and hypertrophy, but the addition of new nephrons has not been observed so far [14]. Another remarkable example of compensatory growth by hyperplasia is the removal of blastomeres in morulae-stage embryos, as this is fully compensated by the embryo. In human preimplantation genetic diagnosis (PGD), up to two blastomeres are removed, but at the blastocyst stage, there is no difference in cell number [23] due to compensatory hyperplasia.

Repair is generally used to unspecifically describe a tissue reaction after suffering damage. This can range from no restoration of the tissue pattern or cell numbers, to partial recovery of function by deposition of connective tissue (e.g., formation of scar after myocardial infarction) and to partial restoration of tissue structure, cell number, and function (e.g., skin wound healing).

In summary, a valid definition of regeneration is the reconstitution of the same cell number and pattern/form of the damaged or lost structure, while compensatory growth restores the function, but not necessarily the original pattern and cell number of the structure.

## Regeneration in the heart

The remarkable regenerative capacity of zebrafish is also observed in the heart [6]. After resection of up to 25% of the ventricle, the remaining resident CMs start to proliferate and replace the lost myocardium over a course of 30 to 60 days [35, 37]. A similar observation was made when using cryoinfarction as a lesion model, after which a collagen scar formed and was replaced over a time course of 130 days [24]. However, in both cases, the original shape of the left ventricle was not restored.

**Fig. 1:**
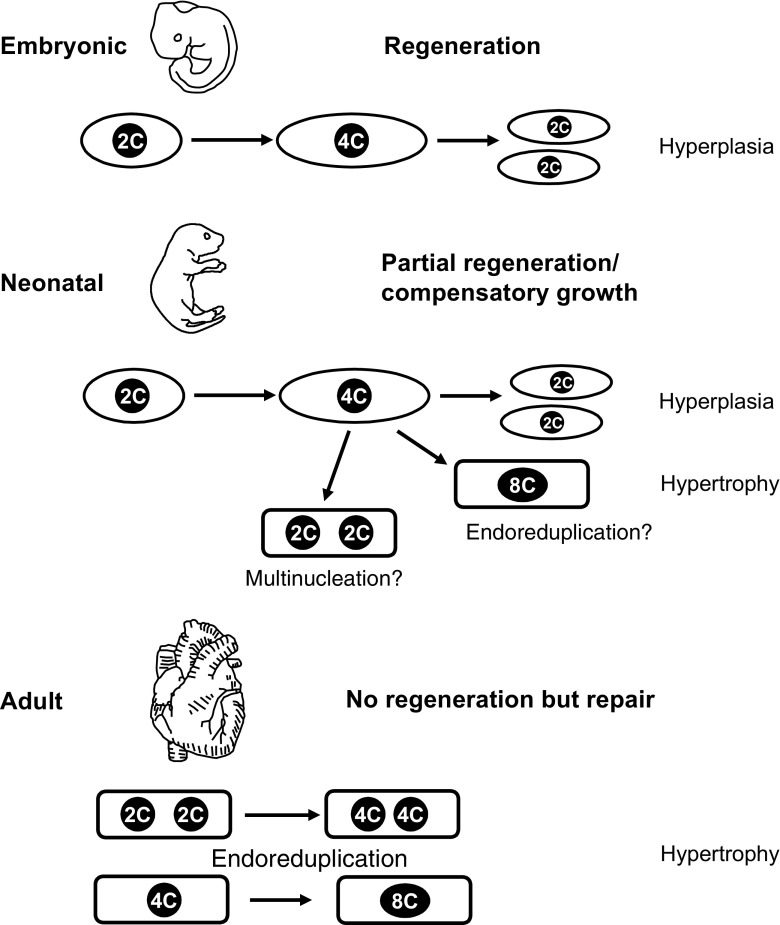
The cell cycle in cardiac regeneration. Cardiac plasticity after injury depends on the developmental stage (e.g., embryonic, neonatal, or adult). Embryonic lesions regenerate by CM division (structure and cell number restored). Lesions in neonatal hearts cause CM cell cycle entry and hyperplasia leading to compensatory growth, as the original structure is not fully restored. It is unclear if endoreduplication or binucleation plays a role in the response. After lesions in adult hearts, neither structure nor cell numbers are restored. CMs enter the cell cycle and undergo endoreduplication leading to hypertrophy

In mammals, the nature of the cardiac repair response to injury differs according to the developmental stage (e.g., embryonic, neonatal, or adult; Fig. 1). While embryonic wound healing of skin has been extensively studied [15, 60], there have been only two reports dealing with in utero embryonic heart lesions and regeneration. Both studies have used genetic models for selective cell deletion of CMs. Drenckhahn et al. genetically depleted mitochondrial energy generation in 50% of all CMs during embryonic development, thereby impairing their function and observed regeneration of almost 80% of these CMs by hyperplasia of remaining CMs [18]. However, as 20% of the impaired CMs survived, 13% of the adult mice displayed dilated cardiomyopathy (DCM), and 40% had pathologies in their cardiac conduction system, proving compensatory growth, but no full regeneration. The more stringent system described by Wu and colleagues deleted cells by overexpression of diphtheria toxin in either Nkx2.5 or αMHC expressing cells [70]. As this strategy affects all CMs during heart development, chimaeras from wild-type and mutant embryos were generated to get a random mixture of affected and unaffected cells. Statistical analysis of organ chimaerism of surviving mice revealed that a loss of up to 60% of cardiac progenitor cells at day E7.5 or a loss of up to 50–60% of CMs at day E9.0 can be compensated for by hyperplasia of the remaining unaffected wild-type cardiac progenitors or CMs, respectively. Follow-up examination did not reveal any gross morphological or functional changes in adult hearts, thereby signifying full reconstitution of organ structure and cell number, providing an example of complete embryonic heart regeneration even upon substantial deletion of embryonic precursors or CMs.

Some of this remarkable regenerative capacity is still active after birth, as neonatal rats displayed myocardial regeneration after apical heart injury [62] with minimal scar formation. In mice, almost complete regeneration in neonatal hearts after resection of a part of the left ventricle has been reported using different injury types [34, 55]. As illustrated by fate mapping experiments, the neomyogenesis is caused predominantly by proliferation of preexisting CMs throughout the heart; in addition, a small degree of contribution by c-kit expressing progenitor cells has been proposed, too [31, 34]. This regenerative ability of the heart is restricted to the first week after birth, as lesions at P7 or later cause scarring and no CM proliferation [55]. Thorough additional analysis revealed a robust induction of cell cycle activity in CMs resulting in CM division, but also formation of a small scar [11]. Follow-up studies of resected mice after 6 months revealed scarring and DCM [4]. Obviously, resection of the cardiac apex in mice leads to compensatory growth, as the original structure is not completely restored. In other lesion models of the neonatal mouse heart, such as cryoinjury and occlusion of the left anterior descending coronary aorta (LAD), neomyogenesis has also been described [25, 34], but appears to be dependent on lesion size [16, 26]. As a mechanism for neomyogenesis, the proliferation of resident CMs was demonstrated [5, 26, 40, 56]. Most interestingly, not only CMs in the border zone of the infarct displayed cell cycle activity, but also CMs in remote regions, which implies some sort of signaling and activation throughout the heart. It is still unknown if the increased cell cycle activity in CMs also leads to endoreduplication or multinucleation, besides cell division (Fig. 1). Do these phenomena observed after LAD ligation in neonatal hearts fulfill the criteria of complete regeneration? As after apical resection, a small scar is observed; hence, it is more likely an incomplete regeneration/compensatory growth.

A completely different picture is seen in the adult heart. Ischemic events, such as myocardial infarction, lead to a permanent loss of CMs and replacement of contractile substrate by a collagen containing scar. This process of repair is accompanied by an inflammatory reaction and angiogenesis [74]. The formation of a scar is essential, as dead myocardium is prone to rupture, which would cause instant death. Cardiac repair in adults is characterized by hypertrophy, but the original structure is not reformed.

In conclusion, complete cardiac regeneration after lesion is only observed in the zebrafish heart and the mammalian embryonic heart. In the mammalian neonatal heart, regeneration is incomplete and/or due to compensatory growth, while in the adult heart, a repair process takes place without or neglectable CM proliferation, but hypertrophy (Fig. 1).

## Role of the cell cycle in cardiac regeneration

During postnatal heart growth, CM cell cycle activity starts to diminish as the expression of pro-mitotic factors such as cyclins and cyclin-dependent kinases gets downregulated [36, 69]. Conversely, cyclin inhibitors such as p21, p27, and p57 get upregulated and promote cell cycle exit of CMs [54, 71]. At the same time, the hypertrophic growth phase of the heart takes place, which is characterized by cell cycle variants in CMs. The two most common variants are endoreduplication and acytokinetic mitosis [32].

Endoreduplication is defined as mitosis without karyokinesis or cytokinesis, leading to polyploid CMs, while acytokinetic mitosis lacks cytokinesis, giving raise to multinuclear and mostly binuclear cells. In mice, CM cell division ceases around P3-P5, followed by cell cycle activity which is exclusively due to cell cycle variants leading to hypertrophic heart growth [69]. As early as P14, the majority of CMs in the ventricles of the mouse heart is binuclear [59, 69] with ~ 45% of the ventricular nuclei being polyploid with a DNA content > 2C per nucleus [75]. In humans, around 25% of CMs become binuclear, and 66% of the nuclei will be polyploid [9, 49].

Adult CMs seem to have withdrawn from the cell cycle, as cell cycle activity is a rare event [8, 68] and are therefore considered postmitotic. This was corroborated by epigenomic analysis which revealed repression of cell cycle genes in CMs during preadolescence which is not reverted after cardiac injury [58].

However, cell cycle activity can be observed in CMs after cardiac lesions in the border zone, but this leads most likely to increased polyploidization and/or multinucleation, but not authentic cell division [30, 32, 48]. This could be due to a loss of contractility of individual cells that undergo cell division. Dividing CMs break down their sarcomeres during M-phase to overcome steric hindrance of cytokinesis [1, 32, 55]. Induction of massive proliferation would therefore most likely impair contractile function of the myocardium and further decrease the already lower ejection fraction after a myocardial infarction. Cell cycle variants, such as endoreduplication, on the other hand lead to hypertrophy of CMs and increase of contractile function without the need of decreasing contractility first. Cell cycle activity has been observed in border zone CMs (after lesion). This did not lead to cell division, but rather to endoreduplication and hypertrophy (Hesse et al. 2012). Other reports claimed significant regeneration (~ 30% of border zone CMs) by most likely self-depleting cardiac progenitor cells [33] and/or proliferation of 3.2% of the border zone CMs, which were preferentially mononuclear and diploid [65]. From this data, it is evident that the adult mouse heart loses its plasticity, as was also reported for humans [8, 9, 49].

In summary, during the repair processes taking place in the adult heart after myocardial loss, the majority of CMs entering the cell cycle undergo cell cycle variants, but do not divide.

## Role of the Hippo pathway in cardiac regeneration

One of the major determinants of heart growth during development and early postnatal stage is the Hippo signaling pathway. Inhibition of Hippo signaling is essential for CM proliferation and heart size [28, 76]. Through the phosphorylation of its downstream factor YAP/TAZ, Hippo inhibits Wnt-signaling target genes [28]. It was further demonstrated that CM proliferation is induced by YAP activity, acting through IGF and Wnt-signaling pathways [76]. Consequently, embryonic overexpression of an active form of YAP induces CM hyperproliferation and embryonic lethality due to overgrowth of the ventricles with loss of pump function [76]. This pathway was brought into context with neonatal heart regeneration in mice. Inhibition of the Hippo pathway prolongs the time window in which regeneration after resection of the apex is still possible [27]. Also, inhibition of dystroglycan 1 by agrin [6] or impairment of the dystrophin glycoprotein complex [50] leads to increased YAP activity and prolongation of the regenerative window. In both reports, neomyogenesis was even reported in adult hearts after induction of myocardial infarction by occlusion of the LAD [6] or after transverse aortic constriction [50].

Another factor claimed to play an important role in cell cycle induction is the homeobox gene Meis1, which induces expression of p21 and thereby blocks cell cycle activity in CMs [47]. Deletion of this factor was reported to augment regeneration in neonates beyond P7 and also in adult mice after myocardial infarction, but its expression pattern was put into question by another study [3]. Also, deletion of either p21 or p27 or both does not induce CM proliferation, but enhances tumorigenesis in the compound knockout [22]. However, RNAi knockdown experiments in vitro have shown activation of CM proliferation to some degree [17]. In conclusion, cell cycle activity is observed in CMs after cardiac lesion, but this leads only in neonatal hearts to significant cell division, while adult CMs conduct mainly cell cycle variants and divide only to a small extent [65].

## Induction of cell division in CMs as a strategy for heart regeneration

The observation of cardiac regeneration made in lower organisms such as zebrafish as well as the neonatal heart of mice suggests that in principal, a strategy based on inducing CM proliferation can theoretically succeed [20]. While vessels and fibroblast show a relatively high turnover and remarkable proliferative capacity in human hearts [9], CMs are definitely the limiting factor for compensatory growth or regeneration after injury. Accordingly, as a therapeutic approach, several attempts have been made to induce cell cycle reentry and cell division in adult, differentiated CMs. Among them are genetic manipulations such as CM-specific overexpression of cyclin D2 [53] or cyclin A2 [12], which both cause CM division and partial regeneration after cardiac lesion, as a ~ 20% decrease in infarct size over a time course of 3 to 5 months was observed.

Also, treatment with a variety of substances has been described to enhance regeneration after cardiac lesion by induction of CM proliferation; e.g., application of FGF1 and simultaneous inhibition of p38 kinase [19], application of PPARδ [45], stimulation with periostin (Kühn et al. 2007), which could not be confirmed by several independent groups, or treatment with neuregulin [10], which was also challenged [61]. Other approaches target micro-RNAs, e.g., inhibition of the miR-15 family, which negatively regulates cell cycle genes, led to CM proliferation in adult mice after myocardial infarction [56]. Also, an injection of either miR-199 or miR-590 into the lesioned myocardium of mice reduces infarct size by CM proliferation [43].

Recently, manipulations of the Hippo pathway were implemented as an approach to induce CM proliferation and regeneration after lesion in adult mouse hearts. The inhibition of this pathway by deleting Salvador, a positive regulator of Lats kinases which are negative regulators of the transcriptional co-activators Yap/Taz [27] or overexpression of the downstream effector Yap, resulted in increased CM proliferation [44] and compensatory growth after lesion in the adult heart. The same effect was described after overexpression of the microRNA cluster miR302-367, which was shown to repress the Hippo pathway [73].

A more simple approach was followed in a recent report claiming CM proliferation to be induced by putting mice into a hypoxic environment [52]. This experiment was based on previous work by this group, which demonstrated the importance of hypoxic niches for regeneration in the neonatal heart and that the switch to an oxygen rich environment by 1 week of age marks the end of the regenerative window [57]. Hypoxia is usually used to induce pulmonary hypertension and CM hypertrophy in the right ventricle of adult mice. As left ventricular CM hyperplasia has never been reported in these studies, the findings need to be confirmed by other groups.

All these studies claim induction of authentic cell division in preexisting CMs and significant (up to 60% smaller infarct size) regeneration of the adult myocardium after cardiac lesion. Accordingly, the verification of authentic cell division is of crucial importance. Unfortunately, in most studies, CM cell division is not directly shown, but indirectly proven by EdU, Ki-67, pHH3, or Aurora B-kinase. As EdU indicates DNA-synthesis and Ki-67 is a marker for general cell cycle activity, neither can distinguish authentic cell division from cell cycle variants. Detection of pHH3 is indicative for M-phase, but unable to distinguish between cell division and acytokinetic mitosis. Also, Aurora B-kinase which is used as a kind of “gold standard” for cell division is not an unequivocal marker due to the same reason [42]. Alternatively, some studies use Langendorff-isolation of CMs and counting in a chamber, which is error-prone, due to variabilities in the dissociation procedure and cell survival [42]. Another indirect measure of CM proliferation is increased heart weight with simultaneous exclusion of CM hypertrophy. Usually, cross sections are used to determine CM area as a sign for hypertrophy, but it would be more informative to determine CM volume, as CM hypertrophy is also possible by longitudinal growth [29].

In summary, more sophisticated tools need to be developed to unequivocally demonstrate authentic CM division to be able to test proliferation inducible substances for future clinical application.

## Summary and perspective

Heart regeneration in mammals strongly depends on the developmental stage. While embryonic hearts display a complete regeneration after injury, early neonatal hearts still have the ability of neomyogenesis, but do not regenerate completely. During both stages, resident preexisting CM enters the cell cycle and divides to form new myocardium. Adult hearts have an impaired CM proliferative response, and regeneration is mainly caused by hypertrophy. At this stage, cell cycle active CMs undergo cell cycle variants and become multinucleated and/or polyploid.

As there is a certain degree of controversial data caused by the difficulties in distinguishing authentic cell division from cell cycle variants, there is a need for genetic systems to monitor and quantify cytokinesis. Several systems have been reported of which the eGFP-anillin system [32] was specifically designed for investigation of cell cycle in CMs, while others such as Fucci do not provide high spatiotemporal resolution during M-phase [63]. Those genetic models are mandatory to be able to screen for proliferation-inducing substances in CMs. Several screens have been performed in the past [21], but mainly on early neonatal CMs or on iPS cell/ES-cell-derived CMs. As these CMs are immature and still in the cell cycle, it is not granted that the substances identified in such screens have the potential to induce authentic cell division in adult CMs. For this reason, more mature CMs which can undergo cell cycle variants (e.g., P6 in mice) need to be used for screens. Several reports [10, 65] have claimed proliferation of mononuclear, diploid CMs during regeneration with neomyogenesis, as this cell population is neither multinuclear nor polyploid. This CM population would be also well suited for a screen for proliferation-inducing substances. In this context, it would also be interesting to investigate if there is a difference in the transcriptome under control conditions and after injury, compared to binuclear CMs. The same holds true for investigating the signaling involved in cytokinesis. What are the factors causing proliferation in remote regions of the neonatal heart after injury? Will those factors, once identified, be able to induce proliferation in the adult heart?

Is it even in principle possible to regenerate the adult heart by inducing proliferation of preexisting mature CMs? The regeneration observed in zebrafish as well as in the embryonic and early neonatal mouse heart proves that this can work. Of course, the route of administration, time window, and dose have to be carefully optimized depending on the chemical nature of the compounds identified. In addition, in vivo studies need to assess, whether left ventricular low output failure and/or arrhythmias are induced upon application of CM proliferation enhancing substances. During such a regenerative approach, also, other cell populations need to proliferate, most importantly vessels for blood supply. Formation of collateral vessels and angiogenesis has been observed after myocardial infarction and will support the newly formed myocardium. On the other hand, the amount of proliferation needs to be controlled by the dose of the component to avoid tumor growth. Despite unresolved questions, recent progress in understanding of endogenous regeneration raises some hope to introduce these concepts into clinical reality in the future. Due to well established revascularisation therapies, in hospital mortality of acute myocardial infarction has decreased remarkably. Nevertheless, long-term survival is clearly affected by the amount of loss of contractile tissue. Therefore, biological strategies to induce autoregeneration of adult ventricular myocardium would indeed perfectly complement surgical and interventional myocardial revascularisation.
